# Understanding Study Drug Discontinuation Through EUCLID

**DOI:** 10.3389/fcvm.2022.947645

**Published:** 2022-07-15

**Authors:** E. Hope Weissler, Hillary Mulder, Frank W. Rockhold, Iris Baumgartner, Lars Norgren, Juuso Blomster, Brian G. Katona, F. Gerry R. Fowkes, Kenneth Mahaffey, Marc Bonaca, Manesh R. Patel, W. Schuyler Jones

**Affiliations:** ^1^School of Medicine, Duke University, Durham, NC, United States; ^2^Department of Medicine, University of Bern, Bern, Switzerland; ^3^Faculty of Medicine and Health, Örebro University, Örebro, Sweden; ^4^Faculty of Medicine, University of Turku, Turku, Finland; ^5^AstraZeneca, Wilmington, DE, United States; ^6^Centre for Global Health Research, Usher Institute, Edinburgh Medical School, University of Edinburgh, Edinburgh, United Kingdom; ^7^School of Medicine, Stanford University, Stanford, CA, United States; ^8^Colorado Prevention Center, School of Medicine, University of Colorado Anschutz Medical Campus, Aurora, CO, United States

**Keywords:** peripheral artery disease (PAD), clinical trial, protocol deviation, disparities (health, antiplatelet therapy)

## Abstract

**Introduction:**

Disparities in the care and outcomes of peripheral artery disease (PAD) have been well-established. In part this is due to disparities in enrollment of PAD trial cohorts. However, less attention has been paid to non-random protocol non-adherence after enrollment, which may lead to inaccurate estimates of treatment effects and reduce generalizability of study results. We aimed to ascertain characteristics associated with premature study drug discontinuation in a PAD cohort.

**Methods:**

Using data from EUCLID (Examining Use of Ticagrelor in Peripheral Artery Disease), factors associated with study drug discontinuation were assessed using univariable and multivariable Cox proportional hazards models with time to study drug discontinuation as the outcome of interest. Relationships between study drug discontinuation and major adverse cardiovascular events (MACE; cardiovascular death, myocardial infarction, ischemic stroke), major adverse limb events (MALE; acute limb ischemia, major amputation, and lower extremity revascularization), and all-cause hospitalization were assessed.

**Results:**

Of 13,842 eligible EUCLID participants, 3,886 (28.1%) prematurely and permanently discontinued study drug over a maximum follow-up of 42 months (annualized rate of 13.2 discontinuations per 100 patient-years). In a multivariable model, premature study drug discontinuation was associated with older age (aHR 1.16, 95%CI 1.14–1.19), eligibility based on prior lower extremity revascularization rather than ABI/TBI criteria (aHR 1.14, 95%CI 1.06–1.23), CLI status (aHR 1.23, 95%CI 1.06–1.42), COPD (aHR 1.36, 95%CI 1.24–1.49), and geographic region. In a multivariable analysis, study drug discontinuation was significantly associated with MACE (aHR 3.27, 95%CI 2.90–3.67, *p* < 0.001), MALE (aHR 1.84, 95%CI 1.63–2.07, *p* < 0.001), and all-cause hospitalization (aHR 2.37, 95%CI 2.21–2.54) following study drug discontinuation.

**Conclusions:**

This analysis of EUCLID demonstrates that premature, permanent discontinuation of study drug is relatively common in more than a quarter of PAD patients, is unevenly distributed based on geography and other baseline characteristics, and is associated with worse outcomes in a clinical trial context. Study teams leading future PAD trials may want to address the possibility of study drug discontinuation prospectively, as a proactive approach may help investigators to maintain study cohort diversity and representativeness without sacrificing power and precision.

## Introduction

Peripheral artery disease (PAD), which affects ≥230 million people worldwide, is understudied (1). Consequently, PAD guidelines are supported by relatively poor-quality evidence. Though much data applied to PAD management comes from sub-analyses of coronary studies, PAD patients constitute a distinct population facing barriers to care and outcomes that differ in both degree and kind compared to those experienced by coronary disease patients (1). Although this realization has led to increased focus on conducting PAD-specific research, difficulties enrolling PAD patients, and especially cohorts representative of PAD patients more broadly, have been documented (2). Less attention has been paid to compliance after enrollment and during follow-up. The validity and generalizability of randomized controlled trials can be affected by poor protocol adherence, among other factors (3). Non-random protocol non-adherence (for instance, higher likelihood of study drug discontinuation in certain patient subgroups) may lead to inaccurate estimates of treatment effects and reduce generalizability of study results (4). It has been well-established that adherence to prescribed medications varies across patients with conditions similar to PAD but it is unclear whether this variability exists in clinical trial contexts, and, if so, what effect it has on study results (5). Therefore, we used data from EUCLID (Examining Use of Ticagrelor in Peripheral Artery Disease) to ascertain characteristics associated with premature study drug discontinuation and its effects on outcomes in order to improve PAD-related research by investigating whether the evidence generated is valid and generalizable to broad PAD populations.

## Methods

EUCLID randomized 13,885 patients to receive clopidogrel (75 mg/day) or ticagrelor (90 mg twice daily) for the prevention of cardiovascular events. Its design characteristics and primary findings have previously been published (6, 7). Patients included in EUCLID were at least 50 years old with symptomatic PAD and had either lower extremity revascularization >30 days before randomization or ankle-brachial index ≤ 0.80 at screening (or toe-brachial index of ≤ 0.60 when ankle vessels were non-compressible). For this analysis, patients enrolled in EUCLID who received at least 1 dose of study drug were divided into those who did and did not prematurely and permanently discontinue study drug. Baseline characteristics including demographics, comorbidities, inclusion criteria, and baseline medication use were described. Reasons for premature study drug discontinuation were also described.

In this *post-hoc* analysis using available clinical trial data, factors associated with study drug discontinuation were assessed using univariable Cox proportional hazards models with time to study drug discontinuation as the outcome of interest. Continuous variables were tested for linearity with respect to the outcome using natural cubic splines with knots at the 5th, 35th, 65^th^, and 95th percentiles; variables found to majorly violate the linearity assumption were evaluated using piece-wise linear splines. A multivariable model was also constructed including all (pre-determined) factors of interest (Supplementary Table 1), including randomized treatment. Candidate covariates were chosen based on clinical experience and prior analyses of the EUCLID dataset (8). Gray's test was used to compare the cumulative incidence of premature study drug discontinuation between study arms with discontinuation due to adverse events considered as a competing risk.

Finally, the relationships between study drug discontinuation and future major adverse cardiovascular events (MACE; cardiovascular death, myocardial infarction, ischemic stroke), major adverse limb events (MALE; acute limb ischemia, major amputation, and lower extremity revascularization), and all-cause hospitalization were assessed using unadjusted and adjusted Cox proportional hazard models with study drug discontinuation as a time-dependent variable. Event rates were calculated as number of events per 100 patient-years of follow-up. Missing data was excluded from analyses throughout, without imputation. In models, patients with data missing from 1 or more variables were excluded. Overall missingness was very low (<5%). SAS v9.4 (Cary, North Carolina) was used for these analyses.

## Results

### Baseline Characteristics and Factors Associated With Study Drug Discontinuation

Of 13,842 eligible EUCLID participants, 3,886 (28.1%) prematurely and permanently discontinued study drug over a maximum follow-up of 42 months (annualized rate of 13.2 discontinuations per 100 patient-years). Participants who discontinued study drug were more likely to be from North America (30.1 vs. 18.8%), and less likely to be from Europe (45.6 vs. 57.2%, Table 1). Participants who discontinued study drug were more likely to have coronary artery disease (33.1 vs. 27.4%) and COPD (15.0 vs. 9.5%) but PAD severity was similar (5.7% CLI and 19.0% asymptomatic in study drug discontinuation group vs. 4.2 and 18.6%, respectively). Finally, there were not marked differences in baseline use of medications between patients who did and did not discontinue study drug. However, among participants who discontinued study drug, 53.6% were randomized to ticagrelor (vs. 46.4% randomized to clopidogrel), while 48.5% of those who did not discontinue were randomized to ticagrelor (vs. 51.5% randomized to clopidogrel). The most common reason for premature study drug discontinuation among patients randomized to ticagrelor was the occurrence of an adverse event (*N* = 937, 45.0% of discontinuations), but the most common reason among patients randomized to clopidogrel was patient decision unrelated to adverse events or endpoints (*N* = 658, 36.5% of discontinuations; Table 2). There were 146 discontinuations overall (3.76% of discontinuations) related to the occurrence of clinical endpoints.

**Table 1:** Baseline characteristics of patients with and without premature, permanent study drug discontinuation.

**Characteristic**	**Discontinued drug (*N* = 3,886)**	**Stayed on drug (*N* = 9,956)**
Age (yrs.), median (IQR)	68 (62–75)	65 (59–72)
Female	1,177 (30.3%)	2,707 (27.2%)
Weight (kg), median (IQR)	76 (65–88)	77 (67–88)
SBP (mmHg), median (IQR)	138 (126–150)	137 (127–148)
DBP (mmHg), median (IQR)	79 (70–82)	80 (70–84)
**Region**		
Asia	396 (10.2%)	1,202 (12.1%)
Central/South America	547 (14.1%)	1,192 (12.0%)
Europe	1,772 (45.6%)	5,695 (57.2%)
North America	1,171 (30.1%)	1,867 (18.8%)
**Inclusion criteria for randomization**		
Previous revascularization	2,340 (60.2%)	5,509 (55.3%)
ABI value, mean (SD)	0.78 (0.24)	0.78 (0.22)
ABI or TBI criteria	1,546 (39.8%)	4,447 (44.7%)
ABI value, mean (SD)	0.62 (0.15)	0.63 (0.15)
TBI value, mean (SD)	0.50 (0.16)	0.53 (0.23)
**Limb symptoms**		
Asymptomatic	740 (19.0%)	1,854 (18.6%)
Mild or moderate claudication	1,931 (49.7%)	5,460 (54.8%)
Severe claudication	992 (25.5%)	2,227 (22.4%)
Chronic limb-threatening ischemia	222 (5.7%)	415 (4.2%)
Pain while at rest	129 (58.1%)	246 (59.3%)
Minor tissue loss	71 (32.0%)	133 (32.0%)
Major tissue loss	22 (9.9%)	36 (8.7%)
Major amputation above the ankle	108 (2.8%)	231 (2.3%)
Minor amputation	190 (4.9%)	412 (4.2%)
eGFR	72.2 (55.7–88.0)	76.4 (61.9–91.8)
**Medical history**		
Stroke	326 (8.4%)	805 (8.1%)
Transient ischemic attack	168 (4.3%)	337 (3.4%)
Coronary artery disease	1,286 (33.1%)	2,731 (27.4%)
Myocardial infarction	777 (20.0%)	1,736 (17.4%)
Diabetes mellitus type I or II	1,608 (41.4%)	3,719 (37.4%)
COPD	582 (15.0%)	948 (9.5%)
CHF	500 (12.9%)	1,424 (14.3%)
Hypertension	3,110 (80.0%)	7,711 (77.5%)
Hyperlipidemia	2,979 (76.7%)	7,468 (75.0%)
Prior PCI	728 (18.7%)	1,441 (14.5%)
Prior CABG	526 (13.5%)	1,002 (10.1%)
**Tobacco use**		
Current	1,184 (30.7%)	3,090 (31.2%)
Former	1,878 (48.7%)	4,633 (46.8%)
Never	794 (20.6%)	2,183 (22.0%)
**Number of vascular beds**		
1	2,029 (52.2%)	5,758 (57.8%)
2	1,387 (35.7%)	3,279 (32.9%)
3	470 (12.1%)	919 (9.2%)
**Medications**		
Aspirin	2,670 (68.7%)	6,575 (66.0%)
Clopidogrel	1,273 (32.8%)	3,185 (32.0%)
Statin	2,818 (72.5%)	7,331 (73.6%)
ACE Inhibitor	1,601 (41.2%)	4,017 (40.3%)
Angiotensin-receptor blocker	1,007 (25.9%)	2,465 (24.8%)
Cilostazol	598 (15.4%)	1,493 (15.0%)
**Randomized treatment**		
Clopidogrel 75 mg QD	1,803 (46.4%)	5,129 (51.5%)
Ticagrelor 90 mg BID	2,083 (53.6%)	4,827 (48.5%)

*ABI, ankle-brachial index; ACE, angiotensin-converting enzyme; CABG, coronary artery bypass grafting; CHF, congestive heart failure; COPD, chronic obstructive pulmonary disease; DBP, diastolic blood pressure; eGFR, estimated glomerular filtration rate; LER, lower extremity revascularization; PCI, percutaneous coronary intervention; SBP, systolic blood pressure; TBI, toe-brachial index*.

**Table 2:** Reasons for premature, permanent study drug discontinuation.

	**Ticagrelor *N* = 2,073 (%)**	**Clopidogrel *N* = 1,803 (%)**
Serious or non-serious adverse event	937 (45.0)	631 (35.0)
Patient decision unrelated to non-serious AE or endpoints	691 (33.2)	658 (36.5)
Clinical endpoint	74 (3.6)	99 (5.5)
Investigator's decision	94 (4.5)	89 (4.9)
Requirement for dual antiplatelet therapy	71 (3.4)	91 (5.0)
Requirement for oral anticoagulant therapy	95 (4.6)	99 (5.5)
Severe non-compliance to study protocol	21 (1.0)	18 (1.0)
Eligibility criteria not met	18 (0.9)	18 (1.0)
Other/missing	65 (3.2)	78 (4.3)

In a multivariable model, premature study drug discontinuation was associated with older age (aHR 1.16, 95%CI 1.14–1.19), eligibility based on prior lower extremity revascularization rather than ABI/TBI criteria (aHR 1.14, 95%CI 1.06–1.23), chronic limb-threatening ischemia status (aHR 1.23, 95%CI 1.06–1.42), and COPD (aHR 1.36, 95%CI 1.24–1.49; Table 3). Region was associated with premature discontinuation, with North American participants most likely to discontinue compared to European participants (aHR 1.45, 95%CI 1.33–1.58), followed by Central/South Americans (aHR 1.25, 95% CI 1.12–1.38). Asian participants were less likely to discontinue (aHR 0.82, 95%CI 0.73–0.93). Participants who used statins at baseline were less likely to discontinue (aHR 0.85, 95%CI 0.77–0.94) while those who were randomized to ticagrelor were more likely to discontinue (aHR 1.22, 95%CI 1.15–1.30). When discontinuation due to adverse events was considered to be a competing risk, there was no longer any difference in premature discontinuation between study arms (*p* = 0.73).

**Table 3:** Factors associated with premature, permanent study drug discontinuation in EUCLID.

	**Univariable**		**Multivariable**	
**Characteristic**	**Hazard ratio (95% CI)**	***p*-value**	**Hazard ratio (95% CI)**	***p*-value**
Age	1.19 (1.16–1.21)	<0.001	1.16 (1.14–1.19)	<0.001
Female	1.15 (1.07–1.23)	<0.001	1.00 (0.92–1.08)	0.966
Region		<0.001		<0.001
Asia vs. Europe	1.07 (0.96–1.19)		0.82 (0.73–0.93)	
Central/South America vs. Europe	1.43 (1.30–1.57)		1.25 (1.12–1.38)	
North America vs. Europe	1.75 (1.63–1.89)		1.45 (1.33–1.58)	
**Weight**				
Weight ≤ 80 kg	0.95 (0.94–0.97)	<0.001	0.96 (0.94–0.98)	<00.001
Weight > 80 kg	1.03 (1.01–1.05)	0.001	1.02 (1.01–1.04)	0.008
**SBP**				
SBP ≤ 130	0.96 (0.94–0.98)	<0.001	0.99 (0.96–1.01)	0.275
SBP > 130	1.03 (1.02–1.04)	<0.001	1.02 (1.01–1.03)	0.003
DBP	0.95 (0.94–0.97)	<0.001	1.00 (0.98–1.02)	0.927
**eGFR**				
eGFR ≤ 75	0.93 (0.92–0.94)	<0.001	0.96 (0.94–0.97)	<0.001
eGFR > 75	1.00 (0.99–1.01)	0.795	1.01 (1.00–1.02)	0.145
**ABI**				
ABI ≤ 0.65	0.93 (0.90–0.97)	<0.001	0.95 (0.91–0.98)	0.006
ABI > 0.65	1.04 (1.02–1.07)	<0.001	1.03 (1.01–1.06)	0.006
Prior LER vs. ABI/TBI inclusion	1.17 (1.10–1.25)	<0.001	1.14 (1.06–1.23)	<0.001
Chronic limb-threatening ischemia	1.35 (1.18–1.55)	<0.001	1.23 (1.06–1.42)	0.005
Prior major amputation	1.17 (0.96–1.41)	0.111	1.05 (0.86–1.29)	0.604
Prior minor amputation	1.19 (1.03–1.38)	0.020	1.15 (0.98–1.33)	0.080
Prior stroke	1.05 (0.94–1.17)	0.417	1.03 (0.91–1.17)	0.638
Prior TIA	1.25 (1.07–1.46)	0.004	1.10 (0.93–1.29)	0.275
Prior MI	1.17 (1.08–1.26)	<0.001	1.07 (0.97–1.19)	0.166
Diabetes	1.16 (1.08–1.23)	<0.001	1.10 (1.03–1.18)	0.006
Hypertension	1.14 (1.05–1.23)	0.001	0.95 (0.86–1.04)	0.289
Hyperlipidemia	1.06 (0.98–1.14)	0.124	1.05 (0.94–1.17)	0.392
CHF	0.93 (0.84–1.02)	0.118	0.90 (0.81–0.99)	0.036
COPD	1.52 (1.39–1.66)	<0.001	1.36 (1.24–1.49)	<0.001
Prior PCI	1.30 (1.20–1.41)	<0.001	1.13 (1.01–1.25)	0.026
Prior CABG	1.32 (1.20–1.45)	<0.001	1.09 (0.97–1.22)	0.136
Number of vascular beds		<0.001		0.940
2 vs. 1	1.17 (1.09–1.26)		1.00 (0.91–1.10)	
3 vs. 1	1.38 (1.25–1.52)		0.98 (0.84–1.15)	
Current smoker	0.97 (0.90–1.04)	0.339	1.11 (1.03–1.20)	0.005
Prior aspirin use	1.11 (1.04–1.19)	0.002	1.02 (0.95–1.10)	0.524
Prior clopidogrel use	1.02 (0.96–1.09)	0.511	0.91 (0.84–0.98)	0.013
statin use	0.94 (0.87–1.01)	0.074	0.85 (0.77–0.94)	0.001
ACE-I/ARB use	1.03 (0.97–1.10)	0.307	0.98 (0.90–1.05)	0.532
Randomized to ticagrelor vs. clopidogrel	–		1.22 (1.15–1.30)	<0.001

*ABI, ankle-brachial index; ACE-I, angiotensin-converting enzyme inhibitors; ARB, angiotensin II receptor blockers; CABG, coronary artery bypass grafting; CHF, congestive heart failure; COPD, chronic obstructive pulmonary disease; DBP, diastolic blood pressure; eGFR, estimated glomerular filtration rate; LER, lower extremity revascularization; MI, myocardial infarction; PCI, percutaneous coronary intervention; SBP, systolic blood pressure; TBI, toe-brachial index; TIA, transient ischemic attack*.

### Clinical Outcomes Associated With Study Drug Discontinuation

Participants who prematurely discontinued study drug had markedly higher rates of clinical outcomes following study drug discontinuation (Table 4). The rate of MACE while on study drug was 3.31 per 100 patient-years (py) vs. 11.59 per 100 py following discontinuation (HR 3.61, 95%CI 3.24–4.02, *p* < 0.001). Within the MACE composite, the risk of cardiovascular death was particularly elevated (1.32/100 py on study drug vs. 5.99/100 py following discontinuation; HR 4.53, 95%CI 3.89–5.28, *p* < 0.001). Similarly, the rate of MALE while on study drug was 5.42 vs. 9.46 per 100 py following discontinuation (HR 1.92, 95%CI 1.72–2.15, *p* < 0.001). Within the MALE composite, the risk of major amputation was particularly elevated (0.46/100 py on study drug vs. 1.66/100 py; HR 4.33, 95%CI 3.27–5.74, *p* < 0.001). All-cause hospitalizations were 20.23 while on study drug vs. 44.70 per 100 py following discontinuation. In a multivariable analysis, study drug discontinuation was significantly associated with future MACE (aHR 3.27, 95%CI 2.90–3.67, *p* < 0.001), MALE (aHR 1.84, 95%CI 1.63–2.07, *p* < 0.001), and all-cause hospitalization (aHR 2.37, 95%CI 2.21–2.54).

**Table 4:** Association between (time-dependent) premature study drug discontinuation and clinical outcomes.

			**Unadjusted**		**Adjusted** ^**a**^	
**Outcome**	**While on study drug rate (events)**	**Not on study drug rate (events)**	**Hazard ratio (95% CI)**	***P*-value**	**Hazard ratio (95% CI)**	***P*-value**
MACE	3.31 (948)	11.59 (535)	3.61 (3.24–4.02)	<0.001	3.27 (2.90–3.67)	<0.001
CV death	1.32 (385)	5.99 (317)	4.53 (3.89–5.28)	<0.001	4.18 (3.55–4.92)	<0.001
MI	1.52 (436)	5.18 (246)	3.56 (3.03–4.18)	<0.001	2.98 (2.50–3.54)	<0.001
Ischemic stroke	0.69 (200)	1.97 (97)	3.05 (2.37–3.91)	<0.001	3.15 (2.42–4.11)	<0.001
MALE	5.42 (1,476)	9.46 (405)	1.92 (1.72–2.15)	<0.001	1.84 (1.63–2.07)	<0.001
ALI	0.59 (171)	1.21 (60)	2.50 (1.85–3.38)	<0.001	2.85 (2.06–3.94)	<0.001
Major amputation	0.46 (133)	1.66 (83)	4.33 (3.27–5.74)	<0.001	3.85 (2.81–5.27)	<0.001
Lower extremity revascularization	4.96 (1,358)	8.69 (377)	1.91 (1.70–2.14)	<0.001	1.78 (1.57–2.02)	<0.001
All-cause hospitalization	20.23 (4,737)	44.70 (1,153)	2.45 (2.30–2.62)	< .001	2.37 (2.21–2.54)	<0.001

*ALI, acute limb ischemia; CV, cardiovascular; LER, lower extremity revascularization; MACE, major adverse cardiovascular events; MALE, major adverse limb events; MI, myocardial infarction. ^a^Please see Supplementary Table 1 for variables used in adjustment*.

## Discussion

This analysis of EUCLID demonstrates that premature, permanent discontinuation of study drug is relatively common in more than a quarter of PAD patients enrolled in EUCLID, is unevenly distributed based on geography and other baseline characteristics, and is associated with worse outcomes in a clinical trial context. These findings have implications for design and interpretation of future studies, especially in PAD and similar conditions.

Many factors associated with study drug discontinuation can be understood intuitively. Older patients, those with prior lower extremity revascularization, and those with CLI may have been more likely to discontinue study drug due to clinical events or the need to start full anticoagulation. The association between COPD and study drug discontinuation has previously been reported and may be due in part to the respiratory side effects of ticagrelor, which were the largest subcategory of adverse event-related ticagrelor discontinuation as reported in the main study results (7, 8). Studies that have examined factors associated with study drug discontinuation in other atherosclerotic conditions have reached some similar findings, including associations between study drug discontinuation and older age, hypertension, and chronic kidney disease (9–11). These patterns may be both directly related to more frequent medication adverse effects experienced by older patients as well as being indirectly influenced by the burden of taking multiple medications multiple times a day, which may lead to more non-adherence and related clinical events (though we did not have data about adherence to other non-study medications in this analysis).

The association between region and study drug discontinuation is more difficult to grasp, as it reflects complex interplays between background patient populations, regional approaches to healthcare delivery, and attitudes surrounding clinical research. The geographic breadth of EUCLID was advantageous insofar as it increased participant diversity along many dimensions (12). However, this geographic diversity as well as data collection constraints limited our ability to understand how social determinants of health may have contributed to study drug discontinuation. There are a number of socioeconomic and healthcare delivery characteristics that are different between North America and Europe, including racial/ethnic diversity, robustness of social safety net supports, and cost of and access to healthcare. Social determinants of health are of particular interest both because prior research suggests they may be related to medication non-adherence outside of clinical trial contexts and because a sub-analysis of the ACCORD BP study found that patients who did not achieve their assigned blood pressure target were more likely to be Black and to be less well-educated (5, 13). Overall, 16.0% of patients in ACCORD BP (all of whom had diabetes mellitus, with unknown prevalence of PAD) did not achieve their assigned blood pressure target, whereas 25.4% of 187,691 patients in a recent analysis of 11 cardiovascular, metabolic, and antihyperglycemic therapy-focused trials discontinued study drug (13, 14). Though the rate of study drug discontinuation was similar to our findings, the latter pooled analysis did not report the prevalence of PAD and reported only sex as an explanatory variable (though adjusted for a number of other baseline characteristics), limiting its applicability to PAD populations (14). Therefore, we feel that the present manuscript fills in some important details about risk factors for study drug discontinuation in a high-risk group of patients.

Non-random study drug discontinuation (along with other non-random protocol deviations) can have significant implications for the interpretation of clinical trial results. Non-random study drug discontinuation can lead to inaccurate or incomplete understanding of the drug's efficacy (3, 4). Though this may be partially addressed using statistical techniques such as per-protocol analysis and “while on treatment” estimands, those methods are not foolproof, may introduce new issues, and will not elucidate efficacy for the patients who discontinued study drug (3, 4). Unlike situations in which clinical trial cohorts are clearly non-representative of broader patient populations, non-random study drug discontinuation can decrease generalizability in unrecognized ways unless sub-analyses are conducted of the specific affected subgroups. At the same time, non-random study drug discontinuation could also affect overall study results, leading to under- or overestimation of treatment effect. Given the issues introduced either by excluding patients perceived to be more likely to discontinue study drug or by relying on statistical approaches to mitigate study drug discontinuation *post-hoc*, investigators may wish to consider additional approaches to reduce premature study drug discontinuation. An analysis of study drug discontinuation in IMPROVE-IT found that an early precipitous decline in study drug adherence was effectively countered by enhanced risk-based monitoring and collaboration with sites to address concerns (9). Utilizing a run-in phase, modifying study drug dosage regimens, improving participant access to study nurses in case of questions, and lowering logistical barriers to obtaining study drug have also been suggested as ways to mitigate premature, permanent study drug discontinuation (15, 16). These features of study design may help reduce reliance on overly restrictive cohort selection criteria or statistical techniques to overcome study drug discontinuation. When these study design modifications cannot be employed, more complete ascertainment of features associated with medication adherence including socioeconomic status, healthcare access and delivery, and functional status/quality of life may improve understanding of how study results might apply to the broader population (17).

There are some limitations to this analysis, including those imposed by what data were collected in EUCLID, as well as the difficulties inherent in understanding a complex issue like study drug discontinuation through secondary analysis. This was an exploratory, *post-hoc* analysis, and a full understanding of study drug discontinuation would likely involve collection of additional factors including socioeconomic status and other social determinants of health. Also, though we were able to show that patients who discontinued study drug ultimately had worse outcomes, we don't know whether those worse outcomes were associated with the stated cause for discontinuation or the discontinuation or treatment itself. Furthermore, because data were collected at discrete time points during follow-up, there was some uncertainty surrounding the sequencing of clinical events and study drug discontinuation that prevented analysis of how clinical events affected study drug discontinuation. This same limitation would also complicate a per-protocol analysis. For these reasons, study drug discontinuation prior to occurrence of clinical events should be interpreted as correlative, not causative. Unfortunately, we were not able to analyze laboratory values such as LDL or hemoglobin A1c that may have shed additional light on the relationship between disease control, drug discontinuation, and clinical events. Finally, EUCLID was an explanatory and confirmatory Phase III efficacy trial that enrolled a selected cohort of PAD patients; protocol deviations in more pragmatic effectiveness studies may be more common and carry different implications for interpretation. However, as the first analysis of this kind applied to patients with PAD, it draws attention to the importance of considering study drug discontinuation especially in this potentially high-risk population. Study teams leading future PAD trials may want to address the possibility of study drug discontinuation prospectively, whether through incorporating adherence-focused features into study design or by pre-specifying formative assessments for drug discontinuation during study conduct to ensure that trials' final results are applicable to the intended population, as outlined above. A proactive approach may help investigators to maintain study cohort diversity and representativeness without sacrificing power and precision.

## Data Availability Statement

The data analyzed in this study is subject to the following licenses/restrictions: the data in this article are from the EUCLID dataset and are not able to be shared. Requests to access these datasets should be directed to schuyler.jones@duke.edu.

## Ethics Statement

The studies involving human participants were reviewed and approved by EUCLID was approved at each participating site. The patients/participants provided their written informed consent to participate in this study.

## Author Contributions

EW, HM, FR, MP, and WJ: conception and design. IB, LN, JB, BK, FF, KM, MB, MP, and WJ: data collection. HM and FR: data analysis. EW, HM, FR, and WJ: manuscript drafting. EW, HM, FR, IB, LN, JB, BK, FF, KM, MB, MP, and WJ: manuscript editing and approval. All authors contributed to the article and approved the submitted version.

## Funding

EUCLID was funded by AstraZeneca (NCT01732822).

## Conflict of Interest

EUCLID was funded by AstraZeneca; AstraZeneca did not fund the analysis in this manuscript, design the analysis, or draft the manuscript, but did approve the manuscript. BK was employed by AstraZeneca. The remaining authors declare that the research was conducted in the absence of any commercial or financial relationships that could be construed as a potential conflict of interest.

## Publisher's Note

All claims expressed in this article are solely those of the authors and do not necessarily represent those of their affiliated organizations, or those of the publisher, the editors and the reviewers. Any product that may be evaluated in this article, or claim that may be made by its manufacturer, is not guaranteed or endorsed by the publisher.
