# Supplementary material for: Understanding Study Drug Discontinuation Through EUCLID
Source: Front Cardiovasc Med. 2022 Jul 15;9:947645. doi: 10.3389/fcvm.2022.947645 (PMC9344128; doi:10.3389/fcvm.2022.947645)
Supplement: Supplementary file 1 [file Table_1.docx]

**Supplemental Table 1: Factors pre-specified for inclusion in explanatory models**

| **Outcome** | **Adjustment Variables** |
| --- | --- |
| All models | age, weight, ABI, eGFR, region, inclusion criteria, chronic limb-threatening ischemia, current smoker, statin use, diabetes, prior PCI, prior clopidogrel, randomized treatment |
| **Additional variables:** |  |
| MACE (CV death, MI, stroke) | sex, diseased vascular beds, prior major amputation, prior minor amputation, prior stroke, prior MI |
| CV Death | sex, prior MI, prior stroke, prior minor amputation, prior major amputation, ARB use |
| MI | sex, prior MI, prior minor amputation, ARB use, prior aspirin use |
| Ischemic Stroke | prior stroke, prior minor amputation |
| MALE (ALI, major amputation, LER) | prior major amputation, prior minor amputation |
| ALI Requiring Hospitalization | ARB use |
| Major Amputation | prior major amputation, prior minor amputation |
| Lower extremity revascularization | prior stroke, prior aspirin |
| All-Cause Hospitalization | diseased vascular beds, prior stroke, prior minor amputation, prior aspirin use |

ARB: Angiotensin II receptor blocker; ALI: Acute limb ischemia; CV: Cardiovascular; LER: Lower extremity revascularization; MACE: Major adverse cardiovascular events; MALE: Major adverse limb events; MI: myocardial infarction
